# Volumetric MRI Markers of Cognitive Impairment in Relapsing-Remitting Multiple Sclerosis: Cerebellar White Matter Loss, Pallidum Atrophy, and Choroid Plexus Enlargement

**DOI:** 10.3390/brainsci16020214

**Published:** 2026-02-11

**Authors:** Weronika Galus, Katarzyna Zawiślak-Fornagiel, Julia Wyszomirska, Oskar Bożek, Daniel Ledwoń, Patrycja Romaniszyn-Kania, Aleksandra Tuszy, Joanna Siuda, Andrzej W. Mitas

**Affiliations:** 1 Department of Neurology, Faculty of Medical Sciences in Katowice, Medical University of Silesia, 40-752 Katowice, Polandjsiuda@sum.edu.pl (J.S.); 2 Department of Psychology, Faculty of Health Sciences in Katowice, Medical University of Silesia, 40-752 Katowice, Poland; jwyszomirska@sum.edu.pl; 3 Department of Radiology and Nuclear Medicine, Faculty of Medical Sciences in Katowice, Medical University of Silesia, 40-752 Katowice, Poland; 4 Faculty of Biomedical Engineering, Silesian University of Technology, 41-800 Zabrze, Poland

**Keywords:** multiple sclerosis, cognitive impairment, quantitative MRI, brain atrophy, choroid plexus, silent progression

## Abstract

Cognitive impairment (CI) is a common and disabling manifestation of multiple sclerosis (MS), yet it remains underdiagnosed in clinical settings. This study aims to identify the volumetric MRI markers of CI in MS patients. A total of 79 MS patients were enrolled; after exclusions, 63 with relapsing-remitting MS (RRMS) and 7 with primary progressive MS were analyzed. All participants underwent neuropsychological testing (CVLT, BVRT, CTT, VFT, VST, and SDMT). Brain volumes were analyzed using FreeSurfer. In RRMS, 59% had CI (35% single-domain, 24% multidomain). Multidomain CI was linked to reduced left cerebellar white matter and bilateral pallidum volumes, slight choroid plexus enlargement, and higher lesion volume versus cognitively preserved patients. Significant correlations were found between brain volumes and cognitive test scores: cerebellar and cerebral white matter, corpus callosum, subcortical gray matter, and thalamus volumes correlated positively with measures of processing speed, memory, and verbal fluency, while higher lesion load and larger choroid plexus volumes were associated with poorer cognitive performance. CI in MS is linked to both global and regional brain atrophy, as well as lesion load. Volumetric MRI, including choroid plexus analysis, may represent candidate imaging correlates of CI; however, longitudinal and externally validated studies are needed to confirm their predictive value and clinical utility.

## 1. Introduction

Multiple sclerosis (MS) is an autoimmune disease of the central nervous system (CNS) that primarily affects young adults, with a rising global prevalence, and is one of the leading causes of non-traumatic neurological disability [1]. Neuroinflammation leads to focal demyelination, diffuse neurodegeneration, and axonal damage, all of which contribute to the progression of disease. Although MS remains an incurable condition, modern treatments—particularly high-efficacy disease-modifying therapies (DMTs)—are effective in delaying disease progression [2]. Classical activity includes clinical relapses with new neurological symptoms and magnetic resonance imaging (MRI) progression (new/enlarging or active lesions). The most common subtype of MS is the relapsing-remitting form (RRMS), which accounts for approximately 85% of cases at onset [3]. Relapse severity and neurological status in patients with MS (pwMS) are assessed using the Expanded Disability Status Scale (EDSS) [4].

Among MS manifestations, cognitive impairment (CI) is one of the most debilitating symptoms, significantly affecting patients’ quality of life [5]. The most prevalent deficit is reduced information processing speed, which is often considered a core feature of CI in MS and is typically assessed using the Symbol Digit Modalities Test (SDMT) [6,7]. Attention and concentration problems are also common, especially in tasks requiring sustained or divided attention [8]. Deficits in verbal learning and working memory frequently occur, particularly affecting the ability to acquire and retain new information, even when long-term memory remains relatively preserved [9]. Executive dysfunction, including impaired planning, cognitive flexibility, and inhibitory control, may further impact daily functioning and autonomy [10]. Verbal fluency, both semantic and phonemic, is often reduced, reflecting disruptions in language production and retrieval processes that are frequently associated with damage to subcortical and cortical regions, such as the thalamus and basal ganglia [11]. These domain-specific impairments highlight the multidimensional nature of CI in MS and underscore the need for comprehensive neuropsychological assessment strategies.

CI affects approximately 34–65% of pwMS and can occur at any stage of the disease, with estimated prevalence ranging from 30 to 45% in RRMS to 50–75% in secondary progressive MS (SPMS) [12], and 60–80% in primary progressive MS (PPMS) [13]. CI in pwMS may also be influenced by the brain’s inherent ability to learn cognitive skills, which contributes to individual variability in cognitive resilience and performance [14]. Recent studies show cognitive decline in pwMS may occur as progression independent of relapse activity (PIRA), driven by chronic neurodegeneration, smoldering inflammation, and progressive brain atrophy [6].

Volumetric measurements of the brain—either globally or within specific CNS anatomical regions—play a crucial role in assessing the progression of neurological disability, including CI in pwMS [15]. Thalamic atrophy is one of the most robust markers of CI and has been shown to strongly correlate with deficits in processing speed, memory, and executive functions [11]. Atrophy of the caudate nucleus, putamen, and globus pallidus has also been implicated in impaired cognition, particularly in relation to attention and information processing speed [16]. The hippocampus is closely linked to deficits in verbal memory and learning [17]. Damage to the frontal and temporal lobes is associated with executive dysfunction, impaired fluency, and disturbances in semantic memory [16,17]. Additionally, regional brain atrophy, particularly in the whole brain, gray matter, cerebellum, insula, and limbic and temporal lobes, is associated with these findings, identified using automated volumetric MRI software validated for clinical use, which supports its potential application in single-patient assessment [18], whereas in a study by Golan et al., computerized cognitive scores were significantly associated with regional brain volumes, particularly thalamic and ventricular measures, supporting their predictive validity in clinical evaluation of MS patients [19]. Moreover, volumetric measurements of other brain structures, such as the choroid plexus, have been significantly associated with longitudinal decline in visuospatial memory, whereas the volume of the choroid plexus showed no significant relationship with other cognitive domains, including processing speed [20].

Although cognitive assessment is an important component of comprehensive care for pwMS [21], it is not routinely implemented in clinical practice, as observed in many countries, including Poland. This is largely due to limited access to neuropsychologists with specific expertise in multiple sclerosis and the time-consuming nature of formal cognitive assessments, particularly those designed to evaluate multidomain profiles of cognitive deficits. As a result, many cases of cognitive dysfunction remain underrecognized [7]. Therefore, identifying reliable radiological markers associated with CI is crucial for identifying patients at higher risk of cognitive decline. Such markers could guide the selection of individuals who would benefit most from in-depth neuropsychological evaluation, allowing for the earlier implementation of targeted cognitive rehabilitation or therapeutic interventions. Accordingly, this study aims to evaluate CI and investigate radiological correlates, specifically overall brain atrophy and atrophy of strategically important structures, in patients with multiple sclerosis.

In contrast to prior volumetric studies focused mainly on thalamic or global atrophy, this work assesses cerebellar white matter, basal ganglia (especially the pallidum), and choroid plexus volume as correlates of cognitive impairment. It combines these measures with comprehensive neuropsychological profiling and quantification of lesion burden.

## 2. Materials and Methods

### 2.1. Material

A total of 79 patients treated in the Department of Neurology and the Neurological Outpatient Clinic of the University Clinical Center, Prof. Gibinski Medical University of Silesia in Katowice, were recruited for the study. All patients provided informed consent to participate in the study. Inclusion criteria were a diagnosis of MS (RRMS, SPMS, or PPMS) according to the 2010 or 2017 McDonald criteria, age ≥ 18 years, EDSS ≤ 6.5, and current treatment with a DMT. Exclusion criteria were relapse within 6 weeks, dementia-level cognitive impairment, EDSS > 6.5, use of non-DMT immunosuppressive/chemotherapeutic agents (e.g., mitoxantrone and cyclophosphamide), pregnancy or breastfeeding, comorbid neurological or psychiatric disorders (including recent worsening of depressive symptoms within 4 weeks), clinically relevant secondary causes of cognitive impairment (e.g., uncompensated hypothyroidism, significant electrolyte disturbances), and any other severe illness.

A detailed medical history and a neurological examination with EDSS assessment were performed at baseline. All patients underwent a neuropsychological examination performed by an experienced and specialized neuropsychologist, and an MRI scan of the head with contrast was obtained on the day of testing whenever possible. If not possible, the scan closest to the assessment date was used, provided it was acquired within a maximum interval of 6 months.

### 2.2. MRI Protocol

All patients underwent annual MRI as part of routine MS monitoring, in line with the recommendations of the Polish Medical Society of Radiology and the Polish Society of Neurology [22]. Examinations were performed using a GE SIGNA Artist 1.5T scanner (GE Healthcare, Chicago, IL, USA) with a 19-channel Head & Neck coil. Only volumetric series were analyzed: T1-weighted MP-RAGE (axial, precontrast) and T2-weighted FLAIR FS CUBE (sagittal). All images were filtered with the SCENIC algorithm and saved in DICOM format; filtered data were used for volumetry. Segmentation and brain atrophy measurements were performed using FreeSurfer v7.4.1 [23], including intracranial volume, gray and white matter, CSF, and bilateral subcortical structures (ventricles, thalamus, hippocampus, cerebellum). A normalization procedure was performed to adjust all regional brain volumes to each subject’s estimated Total Intracranial Volume (eTIV). Additionally, white matter lesion load was quantified using the Lesion Segmentation Toolbox [24].

### 2.3. Cognitive Assessment

We selected neuropsychological assessment tools covering a broad range of cognitive functions, including auditory, visual, and graphomotor tasks. Preference was given to instruments frequently used in previous studies and recommended for assessing cognitive functions in pwMS, with priority for Polish adaptations or standardizations, especially for language-dependent tasks. To identify individuals with impairments in specific domains, raw scores were converted into standardized scores (sten scores, percentiles, and scaled scores). For the California Verbal Learning Test (CVLT) [25], Benton Visual Retention Test (BVRT) [26], Color Trails Test (CTT) [27], and Verbal Fluency Tests (VFT) [28], Polish norms were used, while for the Victoria Stroop Test (VST) [29] and Symbol Digit Modalities Test (SDMT) [30], international norms were applied. Results below average (indicating impairment) were defined according to each test’s scoring guidelines (typically below sten 4, 16th percentile, or scaled score threshold). All information on the tools, scales, variables, and norms is provided below:

1. **California Verbal Learning Test (CVLT)** in the Polish adaptation by Łojek and Stańczak [25]. Norms from the Polish general population, in sten scores—assessing verbal learning, learning and recall strategies, short- and long-term memory, and executive functions (e.g., inhibition, monitoring, and retrieval strategies):
  - Total Recall Trials (TRT): sum of correct responses (words recalled) across the five learning trials of List A (Trials 1–5)—verbal-auditory learning effectiveness;
  - Short-Delay Free Recall (SDFR): number of words freely recalled from List A after an interference list (List B) is presented and recalled—verbal short-term memory and resistance to interference;
  - Long-Delay Free Recall (LDFR): number of words recalled without cues from List A after a 20-minute delay—long-term verbal memory effectiveness;
  - Discrimination Index: ratio of correct recognitions (hits) to false positives—verbal recognition accuracy involving executive functions;
  - Total Errors (TE): intrusions and perseverations in TRT+SDFR+LDFR+SDCR-+LDCR—semantic memory deficits, executive function deficits.
2. **Benton Visual Retention Test (BVRT)** in version C in accordance with method A in the Polish adaptation by Jaworska et al. [26]. Norms from the Polish general population, in sten scores, are classified into three qualitative categories based on raw score cut-off points—assessing short-term visual memory, visual perception, and visual-constructive abilities through the reproduction of geometric figures:
  - Number of Correct Reproduction (CR): number of fully accurate reproductions of entire figures—visual-perceptual accuracy and visual organization, short-term visual memory efficiency, and visuoconstructive abilities;
  - Number of Errors (NE): total count of reproduction errors, including figure or size distortions, omissions, rotations, misplacement, perseverations—deficits in visual perception, visuospatial organization, and executive control (e.g., spatial planning and error monitoring).
3. **Verbal Fluency Tests (VFT)** standardized in Polish by Gawda and Szepietowska [28]. Norms from the Polish general population in sten scores—assessing many cognitive functions simultaneously, including processing speed, executive functions, semantic memory, lexical memory, and grammar:
  - Phonemic Fluency: two 1-min trials requiring generating as many words as possible beginning with the letters K and F—executive functions, especially initiation, strategy use, inhibition, and working memory, processing speed;
  - Semantic Fluency: two 1-min trials requiring the generation of as many words as possible within categories: animals and fruits—semantic memory, lexical access, working memory, strategy use, processing speed;
  - Verb Fluency: one 1-min trial requiring the participant to list as many human actions as possible (e.g., eat, run, write)—lexical memory and grammar, executive function, processing speed;
  - VFT Total Score: Phonemic Fluency + Semantic Fluency + Verb Fluency—processing speed, executive functions, semantic memory, lexical memory, and grammar.
4. **Color Trials Test (CTT)** in the Polish adaptation by Łojek and Stańczak [27]. Norms from the Polish general population, in percentiles—assessing the efficiency of visual attention, processing speed, and executive functions:
  - CTT 1: Time in seconds needed to connect numbered circles (1–25) in sequence—visual attention, field search, perceptual tracking, and processing speed;
  - CTT 2: Time (in seconds) to connect numbers (1–25) in alternating color-number sequence—visual attention, field search, perceptual tracking, sustained and alerting attention, processing speed, and executive functions, particularly cognitive flexibility and task switching;
  - The Interference Index: (CTT2-CTT1)/CTT1—executive functions, the costs of coping with a more difficult task requiring coping with interference.
5. **Victoria Stroop Test (VST)** norms in Scaled Score Equivalents from Troyer et al. [29] based on healthy adults’ results—assessment of processing speed, various aspects of attention, and executive function, especially control and cognitive flexibility, resistance to interference, inhibition, and cognitive control:
  - VST I (dot condition): time in seconds to name the font color of 24 colored circles arranged irregularly—processing speed, perceptual tracking;
  - VST II (word condition): time in seconds needed to name the font color used for 24 neutral words—processing speed, perceptual tracking, selective attention;
  - VST III (interference condition): time in seconds to name the font color of color words, where font color and word meaning are incongruent—resistance to interference, inhibition of automatic reactions, inhibition of automatic responses, cognitive control, set-shifting, executive functions;
  - Interference Index: VSTIII/VSTI—a more precise assessment of executive functioning, specifically the ability to inhibit automatic responses, as it accounts for baseline processing speed. This makes it a more accurate measure of cognitive control, even in individuals who generally perform individual trials quickly.
6. **Symbol Digit Modalities Test (SDMT)** norms in Scaled Score/Means and Standard Deviation Equivalents from Fellows and Schmitter-Edgecombe [30]—assesses processing speed, executive function, working memory, sustained attention, and visuomotor coordination. Sensitive to cognitive slowing in neurological conditions such as multiple sclerosis.

A clinical psychologist conducted and evaluated the neuropsychological assessment under standardized conditions (individually, in a single session, with tests administered in the same order). Additionally, the Beck Depression Inventory-II (BDI-II) [31] was used to assess depressive symptoms and included as a covariate in the statistical analyses.

### 2.4. Statistical Analysis

Clinical characteristics were compared between the RRMS and PPMS groups; however, further analyses included only patients with RRMS. We investigated the relationship between brain volumetrics and cognitive results, age, MS duration, and EDSS, using the Pearson correlation. Group differences were tested using an independent samples *t*-test, Welch’s *t*-test, or Mann–Whitney U, depending on normality (Shapiro–Wilk) and variance homogeneity (Levene’s). Clinical and volumetric measures were also compared across three CI severity groups using ANOVA with Tukey HSD or Kruskal–Wallis with Dunn’s test. Chi-squared tests were used for categorical variables. Partial correlation (Pearson) was used to examine the relationships between brain volumes and cognitive outcomes, controlling for age, education (more than 12 years), and BDI-II.

## 3. Results

### 3.1. Study Group Characteristics

Of the 79 patients with MS meeting the inclusion criteria, we excluded nine from further analysis due to incomplete data. In four subjects, the MRI scan was not performed, and in five subjects, the results of the neuropsychological examination were not complete. Among the remaining 70 subjects, there were 16 males and 54 females. The average age in the study group was 43.7 ± 11.6 years, and the average duration of the disease was 11.7 ± 7.8 years. Among the 70 patients included in the study, 63 were diagnosed with RRMS and 7 with PPMS. Detailed characteristics by MS type are shown in Table 1.

Exploratory analysis suggests PPMS patients were older and had higher EDSS scores than RRMS patients. The mean disease duration was longer in RRMS, with considerable variability (SD), which is suitable for the study’s purpose.

**Figure 1:**
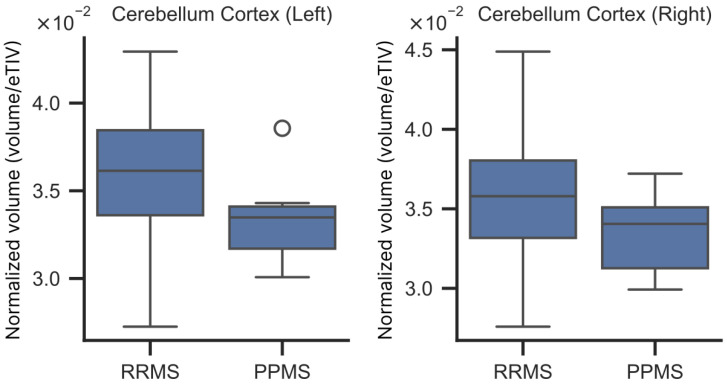
Cerebellum cortex volume normalized by estimated Total Intracranial Volume (eTIV) in patients with relapsing-remitting (RRMS) and primary progressive multiple sclerosis (PPMS).

When comparing brain atrophy by MS type, the cerebellar cortex showed a trend toward lower volume in PPMS vs. RRMS (Figure 1). No other brain structure differences were observed.

**Figure 2:**
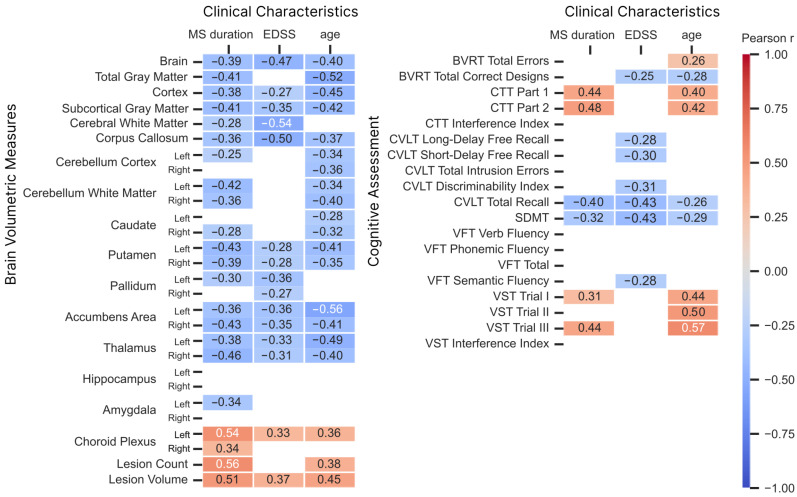
Statistically significant (*p* < 0.05) Pearson correlation coefficients for comparisons between subjects’ clinical characteristics, brain volumetric measures, and cognitive assessment.

In further analysis, we compared clinical characteristics, brain volumetrics, and cognitive results using only patients with RRMS data. Age and MS duration correlated significantly with lesion count/volume and most brain structures, except the hippocampus, pallidum, and amygdala (Figure 2). EDSS scores correlated with total brain volume, cortex, subcortical gray matter, cerebral white matter, corpus callosum, putamen, pallidum, accumbens, and thalamus. Most significant correlations with cognitive scores were common to both age and MS duration, including both CTT parts, CVLT total recall, SDMT, and VST trials I and III. MS duration correlated strongly with age (p<0.001, r=0.53); EDSS showed moderate but significant correlations with age (p=0.019, r=0.29) and MS duration (p=0.013, r=0.31).

Comparisons by gender showed no relationship with specific brain structure volumes. Patients without comorbidities had significantly larger left cerebellum white matter than those with comorbidities (p=0.029, d=−0.59). Cerebellar white matter volume also differed by treatment type, being larger in the high-efficacy treatment group (right: p=0.043, rg=−0.32; left: p=0.045, rg=−0.32).

Several brain structures were significantly larger in the >12 years education group, which was also significantly younger (p=0.016, d=−0.75) and had shorter disease duration (p=0.012, rg=0.45).

### 3.2. Cognitive Impairment Among the Study Group

Among RRMS participants, 26 had all cognitive test scores within the normal range, 22 had one score below normal, and 15 showed impairments on multiple tests (Table 2).

There were no significant differences between cognitive subgroups for sex, age, comorbidities, smoking status, BMI, education level, or treatment (all *p* > 0.05). Age was not linked to CI severity. Disease duration was significantly longer in patients with multidomain impairment than in those with preserved cognition (p=0.011; post hoc: 0 vs. >1, p=0.0087). EDSS scores were also higher in the multidomain group (p=0.002; post hoc: 0 vs. >1, p=0.0014). No other pairwise comparisons were significant.

Volumetric analysis revealed significantly reduced left cerebellar white matter in subjects with multidomain impairment compared to those with preserved cognition (p=0.016). A similar trend was observed on the right side (*p* = 0.018), although pairwise comparisons were not statistically significant. In the basal ganglia, the volumes of the left and right pallidum were significantly lower in patients with multidomain impairment compared to those without (p=0.0028 and p=0.025, respectively). Interestingly, choroid plexus volumes were slightly higher in the multidomain CI group, and a difference was significant for both the left (p=0.015) and right (p=0.034) sides.

**Figure 3:**
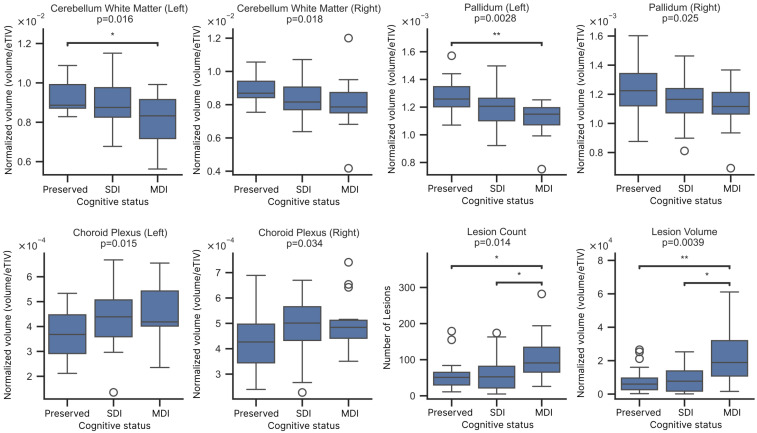
Comparison of selected brain structure volumes and lesion metrics across groups with different levels of cognitive impairment (preserved cognition, single-domain impairment—SDI, and multi-domain impairment—MDI). * p<0.05, ** p<0.01.

For lesion metrics, both lesion count and total lesion volume were significantly higher in participants with multidomain impairment. Lesion count differed among groups (p=0.014), with post hoc tests showing a clear increase in the multidomain group. Lesion volume also differed significantly (p=0.0039), showing a progressive increase in these patients (Figure 3).

### 3.3. Global and Regional Brain Volume in Correlation with Cognitive Performance

**Figure 4:**
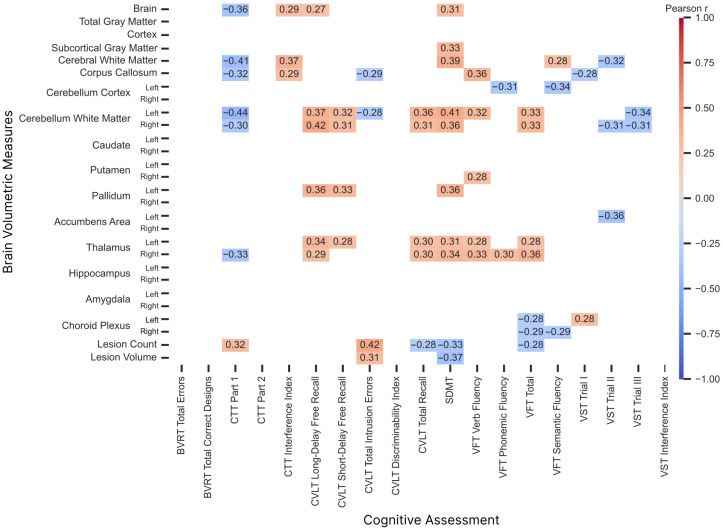
Statistically significant (*p* < 0.05) Pearson correlation coefficients for comparison between brain structure volumes, lesion burden, and cognitive outcomes among patients with relapsing-remitting multiple sclerosis.

The analysis revealed several significant correlations between brain volumes and cognitive test scores, as shown in Figure 4. Among the strongest correlations, cerebellar white matter volumes were negatively correlated with CTT Part 1 (r=−0.44 left, r=−0.30 right) and positively correlated with CVLT Long-Delay Free Recall (r=0.37 left, r=0.42 right), SDMT (r=0.41 left, r=0.36 right), and VFT Total (r=0.33 both sides), whereas left cerebellar cortex volumes showed negative correlations with VFT Phonemic Fluency (r=0.31) and VFT Semantic Fluency (r=0.34).

Similar patterns were observed for cerebral white matter volume, which was negatively correlated with CTT Part 1 (r=−0.41) and positively correlated with the CTT Interference Index (r=0.37) and SDMT (r=0.39).

In line with these findings, corpus callosum volume was negatively correlated with CTT Part 1 (r=−0.32) and positively correlated with VFT Verb Fluency (r=0.36). In the context of subcortical gray matter volume involvement, total subcortical gray matter volume showed a positive correlation with SDMT (r=0.33). Among the subcortical nuclei, thalamus volumes were positively correlated with CVLT Long-Delay Free Recall (r=0.34 left, r=0.29 right), SDMT (r=0.31 left, r=0.34 right), and VFT Total (r=0.28 left, r=0.36 right). Lesion count was positively correlated with CTT Part 1 (r=0.32) and negatively correlated with SDMT (r=−0.33), whereas SDMT scores were negatively correlated with lesion volume (r=−0.37). Choroid plexus volumes showed a negative correlation with VFT Total (r=−0.28 left, r=−0.29 right) and VFT Verb Fluency (r=−0.29 right).

## 4. Discussion

### 4.1. Prevalence and Patterns of Cognitive Impairment Among RRMS Patients

In our RRMS cohort, over half of the patients showed some degree of CI, with about one-quarter having multidomain dysfunction and the rest having single-domain impairment. This pattern aligns with previous studies reporting that 40–70% of MS patients have cognitive deficits [13,32]. The relatively high proportion with preserved cognition may reflect early-stage disease or early treatment initiations.

### 4.2. Radiological Differences Between Preserved Cognition and Multidomain Impairment

Reduced cerebellar white matter volume, especially on the left, may help distinguish patients with preserved cognition from those with multidomain CI, consistent with findings linking cerebellar volume to cognitive performance [33]. Pallidum atrophy was also associated with poorer cognition, consistent with a previous study [34]. Additionally, increased choroid plexus volume may be associated with multidomain CI, possibly reflecting inflammation or altered CSF dynamics [35]. In a large cohort of 1212 people with MS, distinct cognitive phenotypes were associated with specific MRI patterns: mild verbal memory or semantic fluency impairment was linked to smaller hippocampal volume, mild multidomain impairment to reduced cortical gray matter volume, severe executive or attention impairment to higher T2 lesion burden, and severe multidomain impairment to widespread brain atrophy with relative sparing of the pallidum, amygdala, and caudate [36]. Similarly, single-domain impairment is linked to cortical and subcortical atrophy (thalamus, caudate, putamen, accumbens), while significant multidomain impairment involves widespread atrophy sparing mainly white matter and the amygdala [16].

Overall, our findings support that distinct patterns of regional brain atrophy, including cerebellar, pallidal, and choroid plexus changes, may help differentiate cognitive severity in MS. In our study, patients with multidomain CI had higher lesion counts and total lesion volume, supporting the link between greater lesion burden and more severe cognitive decline. Ezzeldin et al. also demonstrated that total and periventricular white matter lesion load are strong predictors of CI in patients with RRMS [37]. However, lesion load alone is less reliable than global measures like brain atrophy, which better reflect cognitive dysfunction in pwMS [38]. Still, focal white matter damage remains a strong predictor of long-term cognitive outcomes [39]. Interestingly, white matter hyperintensities in other neurological conditions are also associated with more severe cognitive deficits, suggesting a shared role of white matter damage in disrupting cognitive networks regardless of underlying disease etiology [40].

### 4.3. Associations of Global and Regional Brain Volume with Cognitive Performance

Taken together, our findings indicate that both global and regional brain volumes are meaningfully associated with cognitive domains in this cohort, which is consistent with evidence from other authors demonstrating that global and regional brain atrophy measures are linked to cognitive deficits [18,41]. Specifically, greater total brain and cerebral white matter volume were consistently linked to better information processing speed, attention, and executive function, as previously reported. This suggests that preserved white matter integrity is crucial for maintaining these functions in neurological conditions [42]. Unexpectedly, the greater volume was associated with a higher cost of executive interference, suggesting compensatory or inflammatory mechanisms in MS, in which increased volume does not necessarily indicate better cognitive function. Such increases may be due to gliosis or edema and may coexist with microstructural damage undetectable by standard MR [43,44].

Additionally, our results suggest that greater corpus callosum volume is associated with better executive functions, including inhibition, cognitive flexibility, and task switching, underscoring the importance of interhemispheric connectivity for cognitive performance in MS. This supports the evidence that corpus callosum volume correlates strongly with cognitive disability [45]. Moreover, the normalized corpus callosum area may serve as a sensitive, practical marker of CI in MS and shows strong correlations with information processing speed [46,47].

Our findings show that larger cerebellar white matter volumes are linked to better processing speed, verbal learning and memory, semantic organization, and executive functions, highlighting the cerebellum’s role beyond motor control in MS. Although cerebellar pathology is common, its impact on cognition remains understudied despite growing evidence of structural and network changes [48]. Earlier studies also show that cerebellar gray matter damage contributes to CI in pwMS [17]. In our study, left cerebellar cortex volume was negatively correlated with lexical retrieval and semantic organization. Verbal fluency, attention, and working memory are often impaired in MS patients with cerebellar lesions [49], reflecting complex cerebellar–cortical interactions in language [50].

In our study, SDMT showed a positive correlation with subcortical gray matter volume. Other studies have also demonstrated that preserved subcortical gray matter volume is associated with better information processing speed, highlighting the key role of deep gray matter structures in cognitive functioning in MS [51].

Whereas atrophy of the left pallidum was associated with both immediate and delayed verbal learning and memory, while pwMS show significant atrophy in the pallidum compared to healthy controls [52], studies do not find a significant correlation between pallidum volume and verbal learning or memory performance. The research instead highlights the thalamus and, to a lesser extent, the hippocampus as more relevant for these cognitive functions [9].

Moreover, our findings show that greater thalamic volume on both sides is associated with better performance in verbal learning and memory, lexical retrieval, processing speed, and executive functions, including cognitive flexibility and strategy. It has been well established that thalamic atrophy in MS, evident even in the early stages, is strongly linked to CI, as the thalamus plays a key role in processing speed, memory, and other functions, thus rendering it a potential biomarker for neuroprotective strategies [53]. Notably, in our study, the right thalamus volume was positively associated with phonemic fluency, whereas the left thalamus was associated with short-term verbal memory and showed negative correlations with attention, processing speed, and field perceptual tracking. In contrast to evenly distributed atrophy, several authors report that predominant atrophy of left-sided thalamic nuclei correlates strongly with worsening cognitive function in pwMS [54]. Moreover, in a detailed assessment of thalamic nuclei atrophy, nuclei closest to the third ventricle—particularly the medial and posterior groups—were more affected, with volume loss in the anterior group being related to CI [11]. Interestingly, the thalamus—as shown in network controllability studies—has a strong influence on cognitive domains related to memory and executive function, which are often assessed using verbal learning tasks in pwMS [10]. Together, these results highlight the thalamus as a key structure across multiple cognitive domains in MS, though the exact mechanisms remain unclear due to disease heterogeneity and individual factors.

Additionally, we found negative correlations between choroid plexus volume and several aspects of cognitive impairment, including lexical and semantic memory, lexical access, executive functions, processing speed, and perceptual tracking. Similarly, another study reported that increased choroid plexus volume was significantly associated with longitudinal decline in visuospatial memory, though it showed no significant correlation with other cognitive domains such as processing speed [20]. Other research suggests that choroid plexus enlargement may contribute to cognitive impairment in pwMS through immune-related mechanisms [35]. Although the mechanism is unclear, it may involve inflammatory or neurodegenerative processes and needs further study. Given the choroid plexus’ role as a blood–CSF barrier structure involved in CSF production and neuroimmune signaling, an important future direction will be to integrate choroid plexus volumetry with CSF biomarker profiles and imaging/physiological measures related to CSF circulation and clearance (e.g., glymphatic and meningeal/dural lymphatic-related dysfunction) to delineate better the mechanisms linking choroid plexus changes to cognitive outcomes in MS [40,55]. In RRMS, choroid plexus enlargement and glymphatic dysfunction have been linked to brain atrophy and greater disability [40]. Choroid plexus volume is often increased in MS, may precede diagnosis, and is associated with brain atrophy and disease severity, suggesting its potential as a marker for monitoring [56].

### 4.4. Radiological Differences in Brain Atrophy Between RRMS and PPMS

Our analysis revealed a trend toward lower cerebellar cortical volume in PPMS than in RRMS, consistent with previous studies [57]. Moreover, greater cerebral cortex atrophy in PPMS than in RRMS is likely linked to the faster progression of disability observed in PPMS patients, as suggested by recent studies [58]. Other researchers have also shown that PPMS patients have reduced cerebellar white matter volume [59]. Furthermore, in progressive forms of MS, cerebellar atrophy has been shown to be associated with CI [60].

### 4.5. Study Strengths and Limitations

One key strength of this study is the integrated assessment of cerebellar white matter, pallidal volume, and choroid plexus enlargement alongside lesion burden and multidomain cognitive profiles. It extends prior MRI–cognition work in MS and identifies additional volumetric markers warranting longitudinal validation.

This study has several limitations. First, the sample size was relatively small, especially for CI level and MS subtype comparisons (RRMS vs. PPMS), which may limit generalizability. Second, although the neuropsychological battery was comprehensive and partially standardized for the Polish population, some tests relied on international norms. Third, the cross-sectional design precludes conclusions about causality or longitudinal changes; follow-up would clarify these trajectories. Fourth, volumetric MRI provided structural insights but did not include advanced techniques like DTI or fMRI to capture microstructural or connectivity changes. The methods used to link cognitive tests to brain structures may have affected results, as some tests are more sensitive to certain regions. Finally, the subgroup-level observations (including multidomain cognitive impairment and PPMS-related comparisons) should be considered exploratory due to limited sample sizes and thus limited statistical power, while the direction of effects appears consistent with the overall pattern, substantial overlap between groups is evident in the corresponding visualizations. Replication in larger cohorts will be necessary to establish the robustness and generalizability of these subgroup-specific signals.

## 5. Conclusions

This study confirmed a high prevalence of cognitive impairment in pwMS, with over half of the RRMS cohort exhibiting some degree of cognitive dysfunction. Patients with multidomain CI exhibited significantly greater total lesion volume and lesion count, as well as reduced volumes in cerebellar white matter—particularly on the left side—and the pallidum, alongside slightly larger choroid plexus volumes, compared to those with preserved cognition. These radiological changes may serve as potential markers for more severe cognitive deficits. Both global and regional brain atrophy were associated with specific cognitive deficits, with reduced volumes of the total brain, cerebral white matter, corpus callosum, cerebellar white matter, thalamus, and pallidum associated with poorer performance in attention, memory, processing speed, and executive functions, while larger choroid plexus volume was linked to worse outcomes across these cognitive domains.

These findings indicate that CI in pwMS is linked to distinct patterns of brain atrophy involving cortical, subcortical, cerebellar structures, and other regions such as the choroid plexus. While volumetric MRI measures may inform group-level cognitive phenotyping and risk stratification, our cross-sectional data do not establish individual-level predictive or monitoring utility. Longitudinal studies with external validation are needed to determine whether these markers can reliably predict cognitive trajectories and support clinical decision-making in MS.

## Figures and Tables

**Table 1:** General characteristics of the study group according to the type of multiple sclerosis.

	RRMS (n = 63)	PPMS (n = 7)
Sex (male/female), n (%)	15 (23.8%)/48 (76.2%)	1 (14.3%)/6 (85.7%)
Age (years), mean ± SD	42.7 ± 11.6	53.6 ± 7.2
Disease duration (years), mean ± SD	12.0 ± 8.1	9.4 ± 3.8
EDSS, median [min, max]	2.0 [0.0, 4.5]	5.0 [3.0, 6.0]
BDI-II, median [min, max]	5 [3, 19]	8 [0, 48]
Comorbidities, n (%)	27 (42.9%)	3 (42.9%)
DMT (platform/HETA), n (%)	19 (30.2%)/44 (69.8%)	0 (0%)/7 (100%)
Nicotinism, n (%)	6 (9.5%)	0 (0%)
BMI, mean ± SD	25.9 ± 6.1	26.1 ± 4.6
Education >12 years, n (%)	46 (73.0%)	5 (71.4%)

BDI—Beck Depression Inventory; BMI—Body Mass Index; DMT—Disease-Modifying Therapy; EDSS—Expanded Disability Status Scale; PPMS—Primary Progressive Multiple Sclerosis; RRMS—Relapsing-Remitting Multiple Sclerosis; SD—standard deviation.

**Table 2:** General characteristics of the patients with RRMS according to the cognitive profile.

	Preserved Cognition (n = 26)	Single-Domain Impairment (n = 22)	Multidomain Impairment (n = 15)	*p* Value, Effect Size
Sex (male/female), n (%)	5 (19.2%)/21 (80.8%)	4 (18.2%)/18 (81.8%)	6 (40.0%)/9 (60.0%)	0.24
Age (years), mean ± SD	40.2 ± 11.8	43.8 ± 11.4	45.2 ± 11.3	0.15
Disease duration (years), mean ± SD	9.0 ± 7.1	12.1 ± 8.2	17.1 ± 7.5	0.011, $\epsilon^{2}$ = 0.11
EDSS, median [min, max]	2.0 [0.0, 4.5]	2.0 [1.0, 4.5]	3.5 [1.0, 4.5]	0.002, $\epsilon^{2}$ = 0.17
BDI-II, median [min, max]	3 [0, 35]	4.5 [0, 32]	7 [0, 48]	0.38
Comorbidities, n (%)	9 (32.1%)	11 (45.8%)	7 (38.9)	0.53
DMT (platform-/HETA), n (%)	7 (26.9%)/19 (73.1%)	6 (27.3%)/16 (72.7%)	6 (40.0%)/9 (60.0%)	0.63
Nicotinism, n (%)	2 (7.1%)	2 (8.3%)	2 (11.1%)	0.99
BMI, mean ± SD	25.5 ± 6.1	26.0 ± 4.3	26.3 ± 8.4	0.85
Education > 12 years, n (%)	21 (75.0%)	17 (70.8%)	8 (44.4%)	0.12

BDI—Beck Depression Inventory; BMI—Body Mass Index; DMT—Disease-Modifying Therapy; EDSS—Expanded Disability Status Scale; SD—standard deviation.

## Data Availability

The data presented in this study are not publicly available due to privacy restrictions.
